# Longitudinal trajectories and risk factors of insomnia symptoms among Chinese bus drivers

**DOI:** 10.3389/fpubh.2025.1713768

**Published:** 2026-01-14

**Authors:** Jingbo Zhao, Huatao Yang, Qiling Fan, Zijie Ma, Yang Yang, Zicong Guan, Guoxi He

**Affiliations:** 1 Department of Psychology, School of Public Health, Southern Medical University, Guangzhou, China; 2 Mental Health Education and Counseling Center, School of Public Health, Southern Medical University, Guangzhou, China

**Keywords:** insomnia symptoms, insomnia, trajectories, bus drivers, longitudinal survey

## Abstract

**Objectives:**

This study investigated the three-year prevalence and longitudinal trajectories of insomnia symptoms among bus drivers and examined key sociodemographic-health-related factors and psychosocial predictors, with the aim of informing targeted preventive strategies.

**Methods:**

A total of 11,576 bus drivers from 22 companies in Guangdong participated in three online surveys at T1 (August–December 2019), T2 (October–December 2021), and T3 (October–December 2023). The surveys assessed demographics, insomnia symptoms, and psychosocial factors. Two-stage multivariate logistic regression models were employed to examine risk factors associated with adverse trajectories.

**Results:**

Prevalence of insomnia symptoms declined steadily from 12.5% at T1 to 7.8% at T3. Five distinct trajectories were identified: resistance (78.5%), chronic dysfunction (1.9%), delayed dysfunction (4.7%), recovery (9.4%), and relapsing/remitting (5.5%). Sociodemographic and psychosocial distress factors (e.g., age, lower financial status, job burnout, family dysfunction) significantly increased the risk of general insomnia and adverse trajectory membership. Crucially, a history of severe physical illness was a strong determinant of chronic risk, while exercising for 30 min daily was significantly associated with a higher likelihood of recovery.

**Conclusion:**

Our study offers insights into the longitudinal trajectories of insomnia symptoms and their associated risk factors among Chinese bus drivers. These findings carry important implications for designing trajectory-specific occupational health interventions and refining public transportation management strategies.

## Introduction

Insomnia is defined as the frequent and persistent difficulty in initiating or maintaining sleep, accompanied by impaired daytime functioning (1). A high prevalence of insomnia symptoms has been documented among professional drivers, including bus, truck, and taxi drivers (2–5). Insomnia symptoms is a critical contributing factor to traffic accidents (6). Evidence suggests that insomnia symptoms directly impairs cognitive processes (7), alters driving behavior, and increases aggressive tendencies (8), thereby elevating multiple risks to driving safety.

Compared to other professional drivers, bus drivers not only bear the responsibility of maintaining road safety but also play a critical role in safeguarding the lives of a large number of passengers, thus holding greater public safety significance. Because of its high passenger capacity, accidents involving public transportation often lead to more severe property damage and casualties compared to those involving private vehicles (9). However, current research on sleep health among professional drivers remains limited, particularly with respect to studies specifically targeting bus drivers. Therefore, identifying and addressing sleep disorders—particularly insomnia symptoms—within this group is essential for promoting driver health and ensuring the overall safety of public transportation systems.

Driver fatigue and drowsiness are among the leading contributors to road traffic accidents (10), as driving in a drowsy state significantly increases the risk of collisions. There are several factors that have been linked to insomnia symptoms in previous research, including gender (11), financial status (12), history of physical illness (13), number of children (14), marital status (15), working hours (16), exercise duration (17), family functioning (18), life satisfaction (19), and job burnout (20). Crucially, most existing studies have employed cross-sectional designs.

This reliance on cross-sectional data is inadequate. Due to the episodic and fluctuating nature of insomnia symptoms, cross-sectional approaches fail to capture the dynamic course of symptom development and related factors over time (21). Therefore, this study uses data from a three-wave survey to fill the gap in the literature. Notably, the longitudinal data collection period [T1 (2019), T2 (2021), and T3 (2023)] spans the pre-pandemic phase and the prolonged routine control phase of the COVID-19 crisis in China. This design was intentionally timed to assess distinct periods: T1 (2019) represents the pre-pandemic baseline, T2 (2021) captures the initial routine control phase, and T3 (2023) reflects the period following the major resolution of public health restrictions. To accurately interpret the observed insomnia trends, it is essential to consider this complex shifting social context. Official reports from the Guangzhou Transport Planning and Research Institute, Co., Ltd. show that starting from 2019, the average daily passenger volume of conventional urban public transport in Guangzhou dropped significantly from 6.11 million to 2.80 million by 2023 (22–24). Critically, this dramatic and sustained decrease in ridership—and the corresponding reduction in workload and passenger exposure—is officially attributed not solely to the pandemic, but also to the rapid, permanent shift in transport dynamics driven by rail transit, ride-hailing services, shared bicycles, and electric bicycles (25). In this context of public health crisis and industry transformation, this study aims to estimate the prevalence of insomnia symptoms among bus drivers, trace heterogeneous developmental patterns, and investigate the factors—and their temporal dynamics—that influence their long-term sleep health trajectories.

## Methods

### Participants and procedure

This study employed a three-wave repeated cross-sectional design with a nested longitudinal subsample. Cluster sampling was used to select 22 bus companies across Guangdong province. Participants were invited to complete online surveys at three assessment points using So-jump, an online survey platform, via a web link or QR code. The assessment points were as follows: T1 (August to December 2019), T2 (October to December 2021), and T3 (October to December 2023).

The questionnaires were distributed by the bus companies and required real-name completion to collect basic driver information, such as name, ID number, employee number, and contact number. All real-name information was used solely for cross-wave data matching, after which the data were anonymized and maintained in strict confidence. The data matching and cleaning process was as follows: First, data across different waves were matched using the participants’ employee number and contact number; subsequently, duplicates were removed based on ID number and name. Furthermore, invalid responses were excluded based on stringent criteria: completion time of less than 2 s per item, 95% or more of the multiple-choice questions selecting the same option, work tenure exceeding 65 years, and participants whose total score on any questionnaire fell outside three standard deviations (±3 SD) of the corresponding sample mean. Following this rigorous cleaning process, the valid sample rates for T1, T2, and T3 were 84.2, 91.04, and 96.95%, respectively. Ultimately, 11,576 participants who completed all three waves were included in the final statistical analysis.

The study was approved by the Human Research Ethics Committee of South China Normal University (Ethics No. SCNU-PSY-2020-01-001). Electronic informed consent was obtained from all participants via an online survey, with the consent document displayed on the cover page. Participants were assured of the voluntary nature of their participation and the right to withdraw at any time.

### Measures

#### Demographics

Demographic factors were collected using a self-designed questionnaire. Variables included gender (female, male), age, driving years, number of children (none, one, two, three or more), marital status (married, unmarried, divorced/widowed), financial status (higher than normal, normal level, lower than normal), history of severe physical illness (no, yes), working hours (≤8 h, 9–10 h, 11–12 h, ≥13 h), and exercise duration (≥60 min, 30–60 min, ≤30 min, never).

#### Insomnia symptoms

Insomnia symptoms were assessed using the Chinese version of the Insomnia Severity Index (ISI), consisting of seven items rated on a 5-point scale (0–4). The total scores range from 0 to 28, with higher scores reflecting greater insomnia severity. A cutoff score of ≥9 was applied to identify probable insomnia in this study (26). The scale exhibited excellent internal consistency across waves (Cronbach’s *α* = 0.91 at T1, 0.89 at T2, and 0.89 at T3).

#### Psychosocial factors

In light of the occupational characteristics of bus drivers, specific psychosocial factors were analyzed, including family functioning, life satisfaction, and job burnout.

Job burnout at T1 was measured using the Maslach Burnout Inventory–General Survey (MBI–GS). Comprising 15 items, the MBI–GS evaluates three core dimensions: emotional exhaustion (EE), cynicism (CY), and reduced personal accomplishment (PA). A composite burnout index was computed using the weighted average proposed by Kalimo et al. (27), Burnout = 0.4 × EE+0.3 × CY + 0.3 × PABurnout = 0.4 × EE+0.3 × CY + 0.3 × PA. A score greater than 1.50 was adopted as the criterion for burnout (28). The internal consistency of the composite index was acceptable (Cronbach’s *α* = 0.85).

The Chinese version of the Family APGAR (29, 30) was used to assess family function at T2. The scale includes five items rated on a 3-point Likert-type scale (0 = never or rarely, 1 = sometimes, 2 = most or all of the time). Scores range from 1 to 10, with higher scores indicating better family functioning. A total score of ≤6 was adopted as the criterion for family dysfunction. The internal consistency was considered acceptable, with a Cronbach’s *α* of 0.83.

Life satisfaction at T2 was evaluated using the Chinese version of the Satisfaction with Life Scale (SWLS) (31). The SWLS comprises five items rated on a 7-point Likert-type scale (1 = strongly disagree; 7 = strongly agree). Total scores range from 5 to 35, with higher scores indicating greater life satisfaction. A total score of ≤19 was used as the cutoff for dissatisfaction in this study (32, 33). Internal consistency was excellent (Cronbach’s *α* = 0.90).

### Data analysis

All statistical analyses were performed with SPSS version 27.0. A two-tailed significance threshold of *p* < 0.05 was applied for all tests. To compare the prevalence of insomnia symptoms across the three survey waves (T1, T2, and T3), repeated-measures Analysis of Variance (ANOVA) was utilized. Bonferroni adjustment was applied to control the family-wise error rate during multiple comparisons. Participants were categorized based on time-varying insomnia symptoms changes (reaching the cut-off point of 9). Based on previous studies (34), trajectories of insomnia symptoms were classified into five types: (1) Resistance; with no symptoms at T1–T3; (2) Delayed dysfunction, with no symptoms at T1 but onset at T2–T3, or no symptoms at T1–T2 but onset at T3; (3) Chronic dysfunction, with symptoms at all three assessments; (4) Relapsing/remitting, with symptoms absent at T1 and T3 but present at T2, or present at T1 and T3 but absent at T2; (5) Recovery, with symptoms at T1 but remission at T2–T3, or symptoms at T1–T2 but remission at T3.

Multivariate logistic regression analyses were performed to explore the determinants of insomnia symptom onset and developmental patterns. Using the resistance group as the reference, a comparison was made with the non-resistance group, which comprised all other groups, to determine predictors of symptom onset. Using the chronic dysfunction group as the reference, a comparison with the recovery group was conducted to identify the factors that reduce the probability of symptom remission. A two-tailed significance threshold of *p* < 0.05 was applied.

## Results

### Sample characteristics

Among the 11,576 bus company drivers, 11,447 (98.9%) were male. This highly male-dominated structure accurately reflects the general gender distribution of the bus driver population in the studied region’s public transit system. The participants had a mean age of 45.2 years (SD = 5.84), with 94.4% falling within the age range of 35 to 55 years. The average driving experience was 19.52 years (SD = 7.90), with 97.9% reporting driving experience between 2 and 33 years. Further details of the sample are provided in Table 1.

**Table 1:** Sample characteristics (*N* = 11,576).

Characteristic (n)	Category (n)	Insomnia symptoms (%)		
		T1	T2	T3
Gender at T1	Female (129)	17.10	10.90	5.40
	Male (11447)	12.50	10.20	7.80
	χ^2^	2.43	0.55	1.04
Age at T1	20 ≤ Age < 30 (161)	16.10	9.90	5.00
	30 ≤ Age < 40 (1758)	12.30	9.80	7.30
	40 ≤ Age < 50 (6886)	12.00	9.80	7.30
	50 ≤ Age < 60 (2771)	13.80	11.50	9.60
	χ^2^	8.03*	6.55	16.61***
Number of children at T1	None (752)	15.00	11.80	7.80
	One (5968)	13.60	10.80	8.30
	Two (4270)	10.90	9.30	7.30
	More than 3 (586)	10.60	9.20	6.80
	χ^2^	22.40***	9.53*	4.37
Marital status at T1	Married (10360)	12.50	10.10	7.70
	Unmarried (443)	14.90	12.90	8.60
	Divorced/Widowed (773)	11.50	10.70	9.60
	χ^2^	3.00	3.84	4.04
Financial status at T1	Higher than normal (389)	9.00	7.50	6.90
	Normal level (5500)	7.30	8.00	6.60
	Lower than normal (5687)	17.80	12.60	9.10
	χ^2^	288.81***	66.05***	25.21***
History of severe physical illness at T1	No (11261)	12.00	9.90	7.60
	Yes (315)	32.70	23.50	16.20
	χ^2^	120.09***	62.05***	31.50***
Working Hours at T2	Less than or equal to 8 h (4588)	11.00	7.30	6.90
	Between 9 and 10 h (6003)	13.90	12.00	8.40
	Between 11 and 12 h (691)	12.40	14.00	9.40
	More than 13 h (294)	8.80	11.60	6.50
	χ^2^	23.53***	74.93***	11.63**
Exercise duration at T2	Over 60 min (1409)	20.40	22.60	14.10
	Between 30 and 60 min (5316)	13.30	11.00	8.40
	Less than 30 min (3881)	9.50	5.80	5.50
	Never (970)	9.10	5.80	4.90
	χ^2^	127.45***	324.28***	119.53***
Family functioning at T2	Good (6271)	10.00	7.00	6.00
	Disordered (5305)	15.50	14.00	9.90
	χ^2^	77.28***	155.25***	60.85***
Life Satisfaction at T2	Satisfied (9356)	10.50	6.90	6.30
	Dissatisfied (2220)	21.30	24.10	14.10
	χ^2^	190.80***	575.58***	152.54***
Job Burnout at T1	Non-Burnout (6053)	4.30	5.60	4.30
	Burnout (5523)	21.60	15.30	11.70
	χ^2^	788.71***	291.67***	220.49***

*p < 0.05; **p < 0.01; ***p < 0.001. T1 = measured at Time 1; T2 = measured at Time 2.

### Prevalence of insomnia symptoms

**Figure 1:**
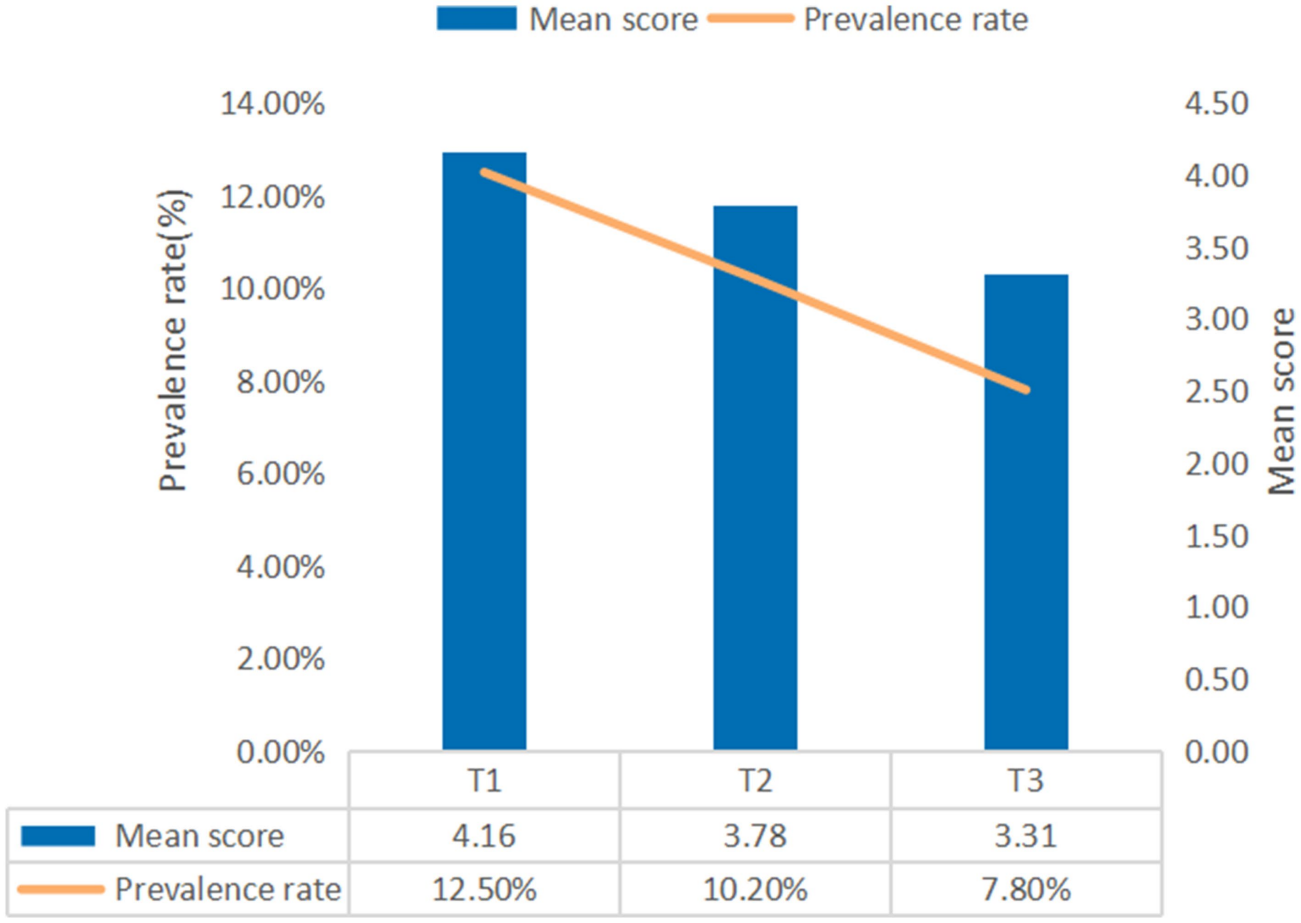
Prevalence rates of insomnia symptoms at the three assessment points. T1 = Time 1; T2 = Time 2; T3 = Time 3.

A progressive decline in the prevalence of insomnia symptoms across the three assessment points is illustrated in Figure 1, with rates of 12.5, 10.2, and 7.8%, respectively. The differences in insomnia symptoms across demographic groups at each time point are presented in Table 1.

### Trajectories of insomnia symptoms

**Figure 2:**
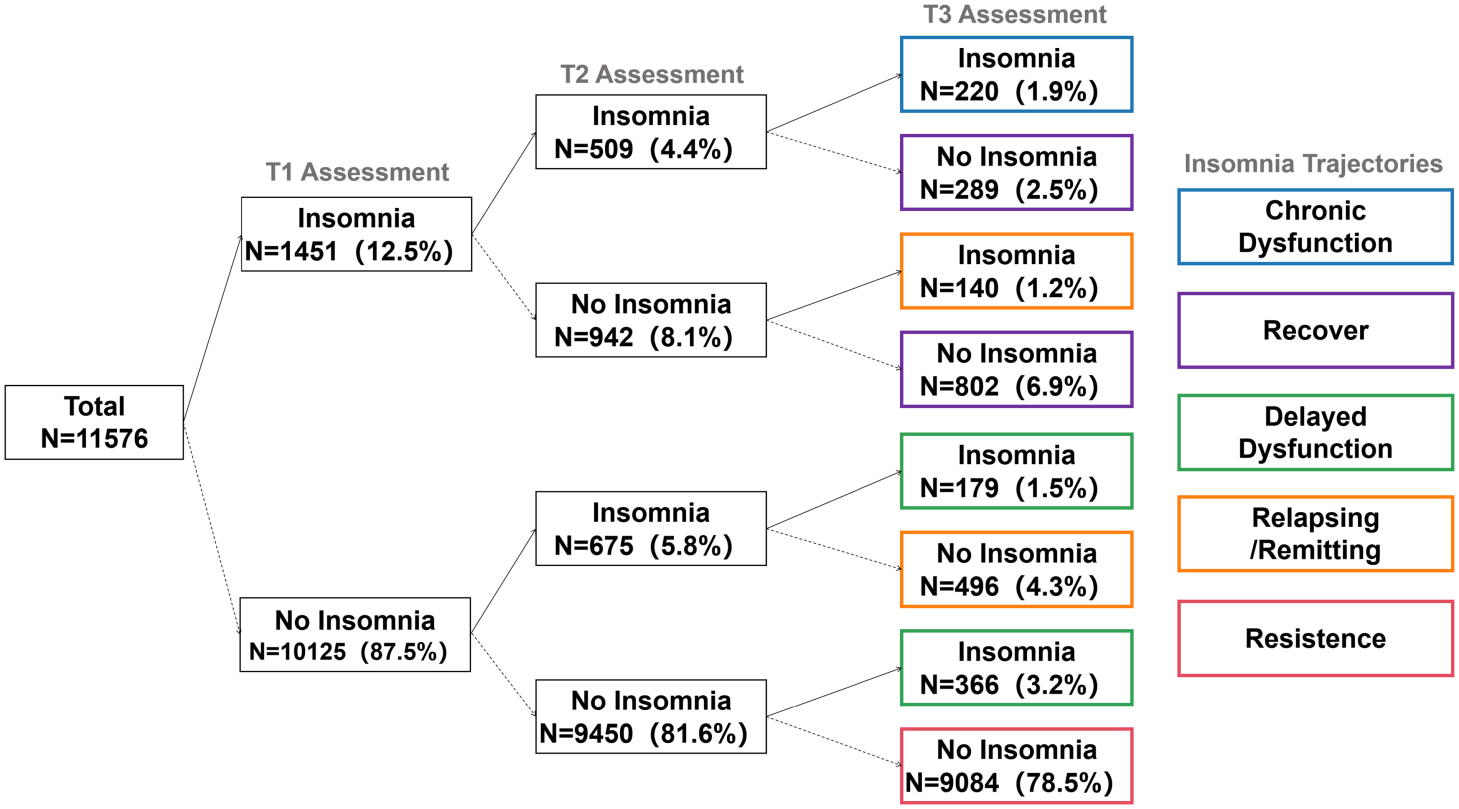
Change patterns of insomnia symptoms. The diagram illustrates the longitudinal flow of insomnia status for the full subsample (*N* = 11,576). Nodes show the number (*N*) and percentage (%) of participants with or without insomnia symptoms at each assessment point. All percentages are based on the total longitudinal sample. The five derived symptom trajectories are defined by their T1-T2-T3 status: Resistance (78.5%): No-No-No. Chronic dysfunction (1.9%): Yes-Yes-Yes. Recovery (9.4%): Yes-Yes-No and Yes-No-No paths. Delayed dysfunction (4.7%): No-Yes-Yes and No-No-Yes paths. Relapsing/remitting (5.5%): No-Yes-No (4.3%) and Yes-No-Yes (1.2%) paths.

**Figure 3:**
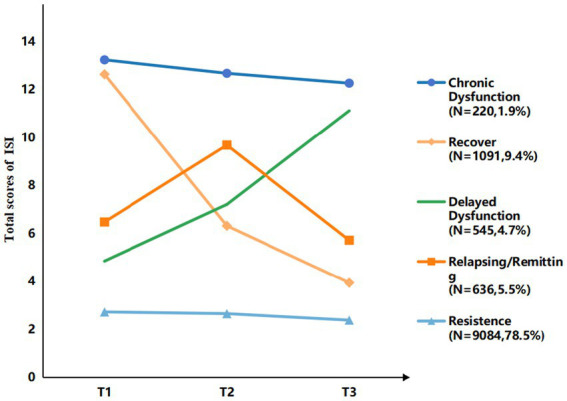
Trajectory of insomnia symptoms. T1 = Time 1; T2 = Time 2; T3 = Time 3.

The change patterns of insomnia symptoms are illustrated in Figure 2, while the five trajectory categories of insomnia symptom trajectories are identified in Figure 3.

The most prevalent group was the Resistance group (No-No-No), comprising 78.5% (n = 9,084) of the drivers who consistently fell below the cutoff for insomnia symptoms across all three assessment points. Conversely, the Chronic Dysfunction group (Yes-Yes-Yes), indicating persistent symptoms, constituted 1.9% (*n* = 220). The Recovery group (Yes-Yes-No or Yes-No-No) accounted for 9.4% of the total sample. This trajectory is defined by participants who met the criteria at T1 and/or T2 but had remitted by T3. The Delayed-Dysfunction group (No-Yes-No or No-No-Yes) accounted for 4.7% of the total sample. This trajectory is defined by participants who were asymptomatic at T1 but met the criteria by T3. The Relapsing/Remitting group totaled 5.5%. This trajectory was formed by two distinct patterns: 4.3% who experienced symptoms at T2 but remitted at T3 (No-Yes-No), and 1.2% who initially showed symptoms at T1, recovered at T2, and relapsed at T3 (Yes-No-Yes).

### Predictors of insomnia trajectory membership

**Figure 4:**
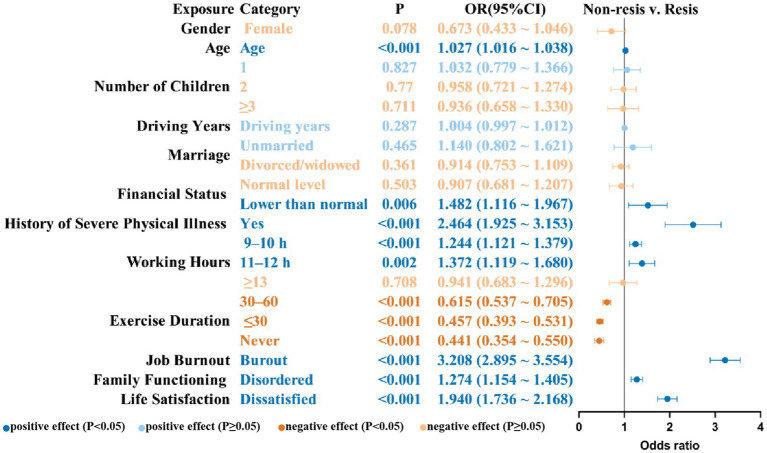
Risk and protective factors associated with membership in the non-resistance group (vs. resistance group). OR, odds ratio; CI, confidence interval; Resis, resistance group; Non-resis, non-resistance group.

Several factors demonstrated significant associations with the occurrence of insomnia symptoms at one or more assessment waves, as illustrated in Figure 4.

These included older age (OR = 1.027, 95% CI = 1.016–1.038), lower financial status (OR = 1.482, 95% CI = 1.116–1.967), a history of severe physical illness (OR = 2.464, 95% CI = 1.925–3.153), working 9–10 h (OR = 1.244, 95% CI = 1.121–1.379) or 11–12 h (OR = 1.372, 95% CI = 1.119–1.680), disordered family functioning (OR = 1.274, 95% CI = 1.154–1.405), low life satisfaction (OR = 1.940, 95% CI = 1.736–2.168), and job burnout (OR = 3.208, 95% CI = 2.895–3.554).

In contrast, shorter durations of exercise were linked to a lower risk of insomnia symptoms when compared to exercising for more than 60 min per day (30–60 min: OR = 0.615, 95% CI = 0.537–0.705; <30 min: OR = 0.457, 95% CI = 0.393–0.531; never: OR = 0.441, 95% CI = 0.354–0.550).

**Figure 5:**
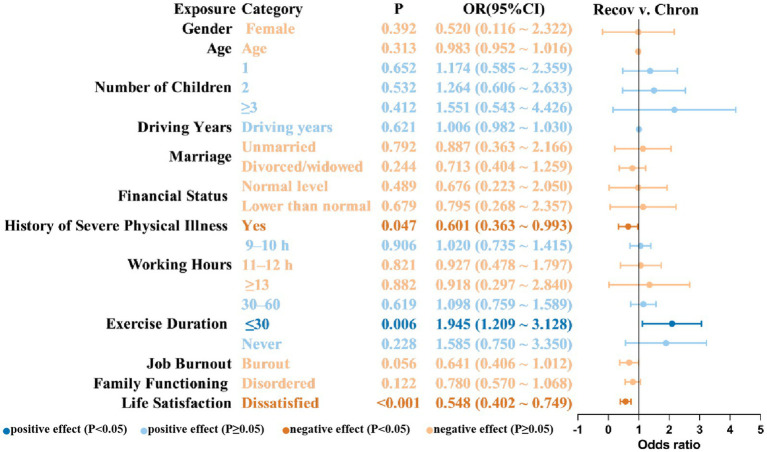
Risk and protective factors associated with membership in the recovery group (vs. chronic dysfunction group). OR, Odds ratio; CI, Confidence interval. Recov, recovery group; Chron, chronic dysfunction group.

As shown in Figure 5, a history of severe physical illness (OR = 0.601, 95% CI = 0.363–0.993) and dissatisfaction with life (OR = 0.548, 95% CI = 0.402–0.749) were found to have a significant impact on reducing the likelihood of being in the recovery group, indicating that these variables may increase the risk of chronic insomnia symptoms. In contrast, shorter exercise durations were significantly related with a higher likelihood of recovery. In contrast, exercising for less than 30 min per day was significantly connected with a higher likelihood of recovery (OR = 1.945, 95% CI = 1.209–3.128).

## Discussion

This study constitutes the first large-scale, multi-wave longitudinal investigation of insomnia symptom trajectories and their predictors among bus drivers. Our findings illuminate the dynamic nature of sleep disturbances within this high-risk occupational group.

From 2019 to 2023, insomnia symptom prevalence exhibited a steady reduction. Five unique trajectories were identified: Resistance, Recovery, Delayed Dysfunction, Chronic Dysfunction, and Relapsing/Remitting. Sociodemographic-health-related factors (age, financial status, history of severe physical illness, working hours, exercise duration) and key psychosocial factors emerged as significant predictors of trajectory membership.

Results showed that the prevalence of insomnia symptoms among bus drivers declined steadily over the three-year period, from 12.5% in 2019 to 10.2% in 2021 and further to 7.8% in 2023, approaching the insomnia rate reported for the general Chinese population (35). This downward trend occurred against the backdrop of the COVID-19 pandemic’s onset and resolution. According to official statistics, bus ridership fell continuously during this interval (22, 23, 25), which may have reduced exposure to passenger aggression (36), improved mental health (37), and consequently benefited sleep outcomes.

Trajectory analysis of insomnia symptoms indicated that, by the 2023 assessment, 78.5% of drivers remained in a no or minimal-insomnia symptoms, reflecting robust adaptation to occupational circadian demands and psychological resilience (38). Similarly, drivers categorized in the Recovery group (9.4%) demonstrated a spontaneous remission after the onset of early insomnia symptoms. In the same time, drivers in the Chronic Dysfunction group (1.9%) experienced persistent insomnia symptoms. Our research focuses on exploring the potential causes of these two trajectories (Recovery and Chronic Dysfunction), which will be discussed in detail below. In contrast, the remaining three trajectories represent patterns requiring clinical attention. The Relapsing/Remitting group (5.5%) was characterized by repeated oscillations in symptom severity Adopting flexible support strategies might be beneficial for improving this situation (39). In the Delayed Dysfunction group (4.7%), drivers showed no insomnia symptoms initially but developed them later, that may reflect the cumulative effects of occupational demands or delayed psychological responses to acute stressors (40). Due to the lack of detailed information regarding drivers’ specific daily routines in this study, we suggest that more targeted monitoring be performed on drivers in this trajectory to identify the precise underlying reasons.

We examine the factors influencing insomnia symptom trajectories among bus drivers and classifies them into two main categories: sociodemographic-health-related factors and psychosocial factors. Increasing age (41), a history of serious physical illness (42, 43), lower financial status (44), and prolonged working hours (9–12 h) emerged as significant predictors of insomnia symptoms among drivers. These findings align with earlier evidence.

Prolonged working hours may lead to insufficient leisure time, subsequently leading to the development of insomnia symptoms. These findings imply that moderately reducing work hours could serve as a protective factor for sleep. However, no significant difference was observed for drivers working more than 13 h per day, possibly due to the small sample size in the ≥13 h group, which reduced statistical power and hindered the detection of an effect. Additionally, it is possible that the group working long hours reflects a healthy worker effect (45), where drivers who are able to engage in long hours of driving may possess better physical and psychological resilience and higher occupational adaptability, thus minimizing the impact of work on their sleep.

Unexpectedly, drivers who exercised for less than 60 min per day or did not exercise at all had a lower risk of insomnia symptoms compared to those who exercised for more than 60 min daily. Additionally, drivers who engaged in regular exercise but limited it to less than 30 min per day were more likely to recover from insomnia symptoms. This phenomenon may be attributed to the fact that the majority of bus drivers work more than 8 h per day, which predisposes them to exercising in the evening. However, evening exercise may hinder sleep onset rather than improve it (46). These results suggest that lifestyle advice for professional drivers should be tailored to their specific situation. It should not be directly applied from general population guidelines. Exercise durations limited to no more than 30 min per day may be more appropriate as a health recommendation for bus drivers or other professional drivers already experiencing insomnia symptoms. It should also be noted that the present analysis did not account for exercise type or habitual patterns (47), which could also be a contributing factor.

Job burnout, family dysfunction, and low life satisfaction are also significant predictors of the onset of insomnia symptoms. Research has shown that prolonged emotional exhaustion and low personal accomplishment in job burnout lead to sustained activation of the hypothalamic–pituitary–adrenal axis (48). This activation increases cortisol levels, contributing to difficulties in falling asleep, sleep maintenance issues, and disruptions in sleep architecture. This may explain why individuals with high burnout scores are more prone to insomnia symptoms. Family dysfunction significantly increases the risk of insomnia symptoms among bus drivers. Family dysfunction often manifests as disharmony and internal conflict, leading to stress and, subsequently, the onset of insomnia symptoms (49). Life satisfaction is the cognitive judgment an individual makes regarding their general quality of life. Life satisfaction is closely related to insomnia symptoms among bus drivers. Low life satisfaction appears to be a salient risk factor for the onset of insomnia symptoms, whereas higher levels of life satisfaction are linked to greater likelihood of recovery. Studies have found that life satisfaction during middle and late adulthood plays a pivotal role in sleep quality during these life stages. Individuals reporting diminished life satisfaction are more prone to heightened anxiety and stress, which are recognized as major contributors to poor sleep quality (50). Higher life satisfaction suggests better subjective physical and mental health, positive lifestyle habits, higher work efficiency, and stronger social relationships (51)—factors that collectively facilitate the alleviation of insomnia symptoms.

Based on these findings, we propose a dynamic model of employee insomnia symptoms during periods of major social change, characterized by declining workload, staff turnover, and reduced sense of meaning. Acute public events and shifts in lifestyle increase workers’ susceptibility to insomnia. However, better physical health (younger age, no major physical illness, regular and appropriate exercise) and a robust social support system (better financial status, life satisfaction, and family functioning) enable workers to either resist insomnia or recover from transient sleep disturbances amidst significant real-life stress.

On the basis of these findings, several practical implications can be drawn. First, targeted screening and multi-component interventions should focus on high-risk drivers, particularly those with older age, severe physical illness, or poor psychosocial health (e.g., job burnout, low life satisfaction). Second, optimizing bus drivers’ work schedules is essential: limiting shifts to a maximum of 8 h is advisable to mitigate adverse sleep effects. Third, lifestyle guidance must be tailored: drivers without symptoms may benefit from 60 min of daily activity, whereas those already experiencing insomnia should maintain exercise durations below 30 min per day for favorable symptom recovery.

The findings of this study should be considered in light of several limitations. Reliance on self-reported measures may have introduced reporting bias. Generalizability is further restricted by the sample structure; it was limited to drivers from Guangdong and was heavily male-dominated (98.9%) due to the profession’s gender-skewed nature, restricting application to female drivers or other occupations. Moreover, potential confounding factors (e.g., exercise types, income changes) were not fully accounted for, and the observational nature of the data precludes definitive causal inference. To address these methodological limitations, future research should employ objective sleep monitoring and comprehensive assessments of occupational environments to explore causal relationships. Interventions should be tailored to distinct insomnia symptom trajectories to enhance treatment effectiveness.

## Conclusion

In conclusion, this study elucidates the dynamic, three-year trajectories of insomnia symptoms among Chinese bus drivers, identifying five distinct patterns. It further demonstrates that sociodemographic-health-related and psychosocial factors associated with their onset and persistence. Additionally, shorter daily exercise durations (≤30 min) appeared to facilitate recovery. These results underscore the necessity of implementing targeted interventions to support the mental health of this crucial workforce. Future research should utilize objective measures to further investigate occupational and lifestyle factors and evaluate the effectiveness of trajectory-specific interventions for improving sleep health and reducing safety-related risks.

## Data Availability

The datasets presented in this article are not readily available because The raw dataset contains personally sensitive information related to participants’ health and psychological status. Therefore, the data are not publicly available. Requests to access the datasets should be directed to mingtian@smu.edu.cn.
